# Assessment of a Peer Support Group Intervention for Undocumented Latinx Immigrants With Kidney Failure

**DOI:** 10.1001/jamanetworkopen.2023.19277

**Published:** 2023-06-21

**Authors:** Lilia Cervantes, Katherine Rizzolo, Kimberly A. Indovina, Claudia Camacho, Cynthia A. Hazel, Xochilt Alamillo, Meghan Chandler, Michel Chonchol, Christine C. Welles, John F. Steiner, Romana Hasnain-Wynia

**Affiliations:** 1 Department of Medicine, University of Colorado, Anschutz Medical Campus, Aurora; 2 Division of Renal Diseases and Hypertension, University of Colorado, Anschutz Medical Campus, Aurora; 3 Department of Medicine, Denver Health and Hospital Authority, Denver, Colorado; 4 Gyedi Project, Denver, Colorado; 5 Denver University Graduate School of Social Work, Denver, Colorado; 6 Division of Nephrology, Denver Health and Hospital Authority, Denver, Colorado; 7 Institute for Health Research, Kaiser Permanente Colorado, Aurora; 8 Office of Research, Denver Health and Hospital Authority, Denver, Colorado

## Abstract

**Importance:**

Most undocumented immigrants with kidney failure rely on emergency dialysis (defined as dialysis after a patient presents as critically ill) and experience significant depression and anxiety and high mortality. Culturally and language-concordant peer support group interventions may be associated with reduced depression and anxiety and may provide emotional support.

**Objective:**

To investigate the feasibility and acceptability of a single-group peer support group intervention.

**Design, setting, and participants:**

This qualitative and single-group prospective study for undocumented immigrants with kidney failure receiving emergency dialysis was conducted in Denver, Colorado, from December 2017 to July 2018. The 6-month intervention included peer support group meetings in the hospital while participants were hospitalized for emergency dialysis. Data were analyzed from March to June 2022.

**Main outcomes and measures:**

To assess feasibility, the recruitment, retention, implementation, and delivery for the intervention were tracked. To measure acceptability, interviews were conducted with participants using a structured format. To assess the value of the peer support group intervention, themes and subthemes were identified from interviews with participants and group meetings.

**Results:**

Of 27 undocumented immigrants with kidney failure receiving emergency dialysis, 23 participants (9 females [39.1%] and 14 males [60.9%]; mean [SD] age, 47 [8] years) agreed to participate (recruitment rate, 85.2%). Among them, 5 individuals withdrew and did not attend meetings and 18 participants (retention rate, 78.3%) attended a mean of 6 of 12 meetings (50.0%). From interviews and meetings, 3 themes, with associated subthemes, were identified: camaraderie and emotional support from peers (subthemes: peer support is vital for people newly diagnosed with kidney failure, safe space to build relationships and share hardship with peers, hospital setting for peer support is ideal, solidarity to survive and change policy, and sustainability of the peer support group), solutions to improve care and resilience (subthemes: self-advocacy, self-motivation and optimism, kidney disease education, emotional support from peers caregivers, and faith), and emotional and physical aspects of receiving emergency dialysis (subthemes: psychosocial and physical distress, mixed experiences with language-concordant care, emotional exhaustion from end-of-life conversations, and gratitude for clinicians).

**Conclusions and relevance:**

This study found that peer support group intervention achieved feasibility and acceptability. The findings suggest that a peer support group may be a patient-centered strategy to build camaraderie and provide emotional support in kidney failure, especially for socially marginalized uninsured populations who report limited English proficiency.

## Key Points

**Question:**

Is a peer support group feasible, acceptable, and of value for undocumented immigrants with kidney failure who rely on emergency dialysis?

**Findings:**

In this qualitative study of 23 undocumented immigrants with kidney failure receiving emergency dialysis, a peer support group intervention had high recruitment, retention, and delivery rates, suggesting it was feasible. Interviews suggested that the intervention was acceptable and valued by participants, who stated that it built camaraderie and a means to share strategies for self-advocacy.

**Meaning:**

These findings suggest that a peer support group may be a patient-centered strategy to provide peer emotional support and camaraderie for undocumented immigrants with kidney failure.

## Teaser

This qualitative study investigates the feasibility and acceptability of a peer support group intervention among undocumented Latinx immigrants with kidney failure.

## Introduction

Latinx individuals experience a 2.1-fold greater incidence of kidney failure than non-Latinx White individuals and face structural racism and discrimination that contribute to kidney health disparities.^1^ Compared with non-Latinx White individuals, Latinx individuals are less likely to receive predialysis nephrology care^2^ or a living donor kidney transplant^3^ or be treated with home dialysis therapies.^4^ Latinx individuals with kidney failure face a disproportionate burden of social challenges, including poverty, lower levels of education, and limited English proficiency.^5,6,7^ People with undocumented US citizenship status (ie, people who entered the US without proper documentation) are primarily Latinx and face additional challenges. Undocumented immigrants are barred from all Medicare health care coverage and the Patient Protection and Affordable Care Act marketplace exchange plans.^8^ In most states, availability of dialysis is limited to emergency dialysis, defined as hospital-based dialysis when the patient is critically ill.^9^ Compared with scheduled, thrice-weekly dialysis, emergency dialysis was found to be associated with a higher hospitalization rate,^10^ 4 times the yearly cost,^11,12^ and 5-fold and 14-fold higher mortality rates at 1 and 5 years, respectively^10^ Undocumented individuals receiving emergency dialysis face significant psychosocial distress associated with the weekly accumulation of symptoms, frequent near-death experiences, unpredictable access to dialysis, and a high level of burden on their caregivers.^13,14,15^

Undocumented immigrants receiving emergency dialysis have reported high levels of depression, anxiety, and reliance on family and peers for support.^6,15,16,17^ Peer support group programs provide patients and their caregivers with social, emotional, and health information support.^18,19,20^ In a systematic review,^19^ more than two-thirds of peer support programs reported improvement in health outcomes. To our knowledge, peer support interventions for undocumented Latinx individuals receiving emergency dialysis have not previously been studied. In this study, we evaluated the feasibility and acceptability of a single-group peer support group intervention during hospitalization for undocumented individuals receiving emergency dialysis.

## Methods

### Study Design

This was a 6-month, single-group, prospective qualitative study of a peer support group intervention. The Colorado Multiple Institutional Review Board (COMIRB) approved this study. All study participants provided written informed consent. We used the Consolidated Criteria for Reporting Qualitative Research (COREQ) reporting guideline.

### Setting and Participants

Eligible participants were adult, undocumented, English- or Spanish-speaking immigrants who self-identified as Latinx/o/a, Hispanic, or both; were diagnosed with kidney failure; and were typically admitted for emergency dialysis on a Tuesday. Race demographics were not collected; only ethnicity was collected given that this was part of inclusion criteria. Institutional policy was to admit these patients overnight every 7 days, and Tuesday was selected for this study because on that day, there were a mean of 14 patients hospitalized for emergency dialysis.

The study lead investigator (L.C.) identified patients, and a culturally and language-concordant research assistant approached participants face to face for study enrollment in December 2017 and January 2018. Participants received $20 compensation for enrollment and $5 for each meeting they attended.

### Peer Support Group Intervention

Participants attended 90-minute support group meetings in a hospital conference room on Tuesday evenings while hospitalized for emergency dialysis. To build trust and *personalismo* (personalized relationships, a Latinx value), meetings had the following structure. (1) The research assistant led meetings. (2) Participants collectively selected topics. (3) Every meeting was started with an icebreaker. (4) A dinner prepared according to a kidney-friendly diet was provided. (5) An optional arts and crafts activity was available. (6) One caregiver per participant was invited. (7) Meeting frequency was weekly for 1 month followed by every 2 weeks for 2 months and then monthly for 3 months. The peer support group intervention lasted 6 months, and there were 12 meetings.

### Outcome Measures and Data Collection

Primary outcomes were feasibility and acceptability, 2 preliminary steps toward the design of an intervention trial. To assess feasibility, we tracked recruitment, retention, and intervention implementation and delivery (ie, frequency, number, and duration of meetings). To measure acceptability, we conducted interviews with participants using a structured format. Meetings were audio-recorded, translated, and transcribed to assess the value of peer support relationships and analyze themes that participants emphasized. Verbatim quotes from meetings and interviews conducted in Spanish were translated to English by a translation company (Datagain). Exploratory measures were collected at baseline and study completion. Anxiety and depression were assessed using the Generalized Anxiety Disorder 7-item scale (GAD-7) and Patient Health Questionnaire 9 (PHQ-9). The GAD-7 includes 7 symptoms of generalized anxiety disorder, and the PHQ-9 includes 9 criteria for depression, with 1 item asking about ideation of suicidal behavior or self-harm. Scores are calculated based on how frequently a person experienced symptoms over the last 2 weeks (eg, a “not at all” response is scored as 0, “several days” as 1, “more than half the days” as 2, and “nearly every day” as 3). The sum of these responses gives a total score. For the PHQ-9, a score of 4 or less is minimal depression, 5 to 14 is mild (5-9) to moderate (10-14) depression, and greater than 14 is moderate severe (15-19) to severe depression (20-27). For the GAD-7, a score of 0 to 4 is minimal anxiety, 5 to 9 is mild anxiety, 10 to 14 is moderate anxiety, and greater than 15 is severe anxiety.

### Data Analysis

Interview transcripts were analyzed by 4 study members (L.C., K.R., K.A.I., and C.H.) of the COMIRB-approved study team using Excel software (Microsoft) from March 1 to June 1, 2022. In this team, 2 members (L.C. and K.A.I.; internal medicine physicians) had previous clinical interactions with participants, while 2 other members (K.R. [nephrology physician] and C.H. [epidemiologist]) did not. Coding and analysis were conducted according to principles of grounded theory and thematic analysis.^21,22^ Line-by-line coding was performed to inductively identify concepts. Similar concepts were grouped into initial themes and subthemes, and then conceptual links among them were identified. Consensus on themes and subthemes was reached after review of the analysis and discussion to ensure that findings reflected the full range and depth of data.^21,22^ Our study was not powered to detect changes in exploratory measures, and we thus provide results at baseline only.

## Results

A total of 23 Latinx adults participated (9 females [39.1%] and 14 males [60.9%]; mean [SD] age, 47 [8] years) among 27 individuals who were approached (Table 1), for a recruitment rate of 85.2%. There were 12 meetings, with a mean (SD) duration of 88 (19) minutes. During the first meeting, participants selected topics they wanted to discuss (eTable in Supplement 1).

**Table 1.** Participant Characteristics

Characteristic	Participants, No. (%) (N = 23)
Demographic	
Age, mean (SD), y	47 (8)
Sex	
Female	9 (39.1)
Male	14 (60.9)
Self-identify as Latinx/o/a or Hispanic	23 (100)
Language preference, Spanish	23 (100)
Preferred meetings in Spanish	23 (100)
Married	12 (52.2)
Socioeconomic	
Education	
<High school	14 (60.9)
High school completion	5 (21.7)
Some college	4 (17.4)
Employed	11 (47.8)
Annual household income, $	
<14 999	16 (69.6)
15 000-29 999	7 (30.4)
Clinical	
Dialysis vintage, mean (SD), mo	44 (34)
Depression at baseline	
Mild	6 (26.1)
Moderate to severe	4 (17.4)
Anxiety at baseline	
Mild	6 (26)
Moderate to severe	4 (17)

### Feasibility and Acceptability

Of 23 participants, 5 participants withdrew prior to meetings and 18 participants (retention rate, 78.3%) attended a mean of 6 meetings (50.0%). Reasons for withdrawing included that the participant moved to Mexico (2 participants), transitioned to a different inpatient dialysis schedule (2 participants), and transitioned to outpatient scheduled dialysis (1 participant). No common patterns of missing meetings were identified. Reasons for missing a meeting included admission to the medical intensive care unit, being on isolation status, a change in the date of weekly hospital admission, and admission to another hospital. We identified 3 themes. The first 2 themes (camaraderie and emotional support from peers and solutions to improve care and resilience) related to the acceptability of the peer support intervention, and the third theme (emotional and physical aspects of receiving emergency dialysis) related to experiences receiving emergency hemodialysis (Table 2**)**.

**Table 2.** Themes, Subthemes, and Illustrative Quotes

Theme or subtheme	Illustrative quote^a^
**Camaraderie and emotional support from peers**	
Peer support is vital for people newly diagnosed with kidney failure	“We must learn to overcome things with each other’s help. I’ve already walked a path others haven’t, and we still have things happening to us. You’re just starting out, but some of us are already weakening, but that’s life. You can learn from our experiences.”
	“Here, we have people who have been doing this for years, and so they can teach us, the ones who are new ones. That can help us…being in communication with these people because we all are suffering from the same things.”
	“Other patients have gone through this for a longer time. It was especially helpful for me when they [other patients] shared their stories.”
	“I would like the program to be offered to more people, especially to newer patients who are just being diagnosed because they could understand better, like myself when I started to understand everything better.”
Safe space to build relationships and share hardship with peers	“Getting to see each other outside the dialysis routine. …you see each other differently and inspire confidence. I feel like the things that I’m going through are the same for others, and that makes us feel better when you get to share with others.”
	“Imagine that you have a big family, lots of friends who…don’t understand why you must come to the hospital every week [for emergency dialysis]. Why do you feel sick? Why do you cry so much? So, for those who’ve had this…medical problem for a long time, how have you communicated with your relatives, your friends, because a lot of people don’t understand.”
	“At first, we didn’t know each other so we were embarrassed to share our stories. And that’s what it’s all about. That’s what I was saying, because we have different stories. Now I know Mr. [deidentified]. … Before, I just said hello. Now, I trust him and that’s what it’s all about.”
	“These persons can tell what lies ahead, because I used to think that with a new kidney you’d have a new life. And all this helps us to mentally focus on the fact that this is a step-by-step process.”
Hospital setting for peer support	“I had the opportunity to be closer to other patients, become friends, share our thoughts and concerns, and be able to help one another…all while in the hospital.”
	“I really liked the hospital meetings. They were perfect because we are here anyways. …we had the chance to get to know each other, share our lived experience. It was a pleasure…especially in my case because my family does not support me. …the support from this group has been amazing.”
Solidarity to survive and change policy	“I created a WhatsApp [messaging application] group, and for our next meeting, we can talk about fundraising by selling cheesecakes.”
	“We can go on TV [television] and talk about how we don’t have health insurance. …we need to work on a press release.”
	“Right now, you’re able to work…but perhaps you won’t be able to in a while. …so if you need something, we can all pitch in. …this is how we do it. …this is how we plant the seed. …we will come here for the hell of it.”
Sustainability of the peer support group	“I’m going to organize a web page with a bit of a mission, the vision, the objectives. …I’ve been sick for a while…but I want to share my thoughts, and if everyone could write a letter about how we feel…we can put this all together.”
	“That’s why I like the idea of having a support group, because if at any given moment we’re working, we’re not here at the hospital, and you need help, you can call or go and meet him and we’ll get together. That’s good because we have to make a support group.”
	“Telling your story, your experience. For example, I created a website, and what we need is story, because someone isn’t going to reach out to a group if they don’t share stories.”
**Solutions to improve care and resilience**	
Self-advocacy	“They’re doctors but you know your own body. And you must take care of yourself, yes? Because I just start crying, and I know I don’t have liquid in my body and I tell them, because I’m the one who’s going to feel sick, not them. So I tell them, ‘That’s enough, buddy!’ I’m crying now. That’s my body and it tells me that way I don’t have more liquid in my body, and so they stop it because if they don’t, I start getting cramps.”
	“When they come in to give me the blood pressure pill and I told the doctor that they should put a big label there indicating they shouldn’t give me that pill because my blood pressure drops. Look, right now. I’m sure it’s above 70/80. I feel really bad and they insist on giving it to me, and I tell the doctor, indicate it there because one of these [days] I’m going to spit it out because I feel I’m going to die.”
	“I think that’s why it’s important to make your own decision. Like me, for example, I’m wearing a purple bracelet. That’s a decision you must make so you won’t let other people decide for you, yes, because my sister, my wife, I want to kill him, no, the decision was mine, I’m the one who doesn’t want this anymore, a box and that’s it. If my heart stops, that’s it. I don’t have to make them decide, the decision.”
	“And you must be aware of everything they do because sometimes you just go, sit down, and they connect you and that’s it, and you don’t ask anything. But you must know what the machine is doing, and you must ask every single thing you have, what is it happening to you, because you just can’t sit there and not know anything.”
Self-motivation and optimism	“And my daughter had the idea, she’d say, ‘I want to die before you do, so I won’t see you’re not going to be there with me.’ And I made the decision because I was about to become, like, depressed, and so I made the decision and I said, ‘I must pull myself together because I don’t want my kids to see me like this.’”
	“I started walking, and I said, ‘I can do it,’ because sometimes you just want to lay down and say you can’t do it. But I started walking and I did feel that I resisted, and then for the next 3 days, I walked really well, and now that I’m back at dialysis, they said my potassium is good. So I said, ‘Well, that’s a good sign.’”
	“I haven’t been doing this for long, but as soon as I started, I looked for information on the internet because when they did dialysis on me I didn’t even know what it was, and a lot of people said I was going to die. But kidney failure isn’t an illness if you don’t want it to be. You can be okay if you want to. You can live a lot of years like this but it’s all to you.”
Kidney disease education	“I told my doctor I wanted to talk to him. …I told him that I didn’t have sex with my husband because I was afraid I could infect him [with my kidney failure], and he laughed at me and told me, ‘How can you infect him? What you have cannot be transmitted.’ So not being well-informed sometimes damages a couple so we need to be informed.”
	“It’s really good to have a good communication with doctors because they’ll guide you while you’re ill. If you don’t have communication with your doctor, how will he know your questions or your pain or what has changed in your diet, food or private life, if you don’t tell him?”
	“If we invite a nutritionist to come and talk to us, this is a safe place. Wherever you are, you can say, ‘I can eat avocadoes.’ What I want to know is how much avocadoes can I eat?”
Emotional support from peers and caregivers	“My wife, my friends, everyone saw me cry because I was torn apart. You’re not made of steel. All of these people, my respect, I went through it, eh? But you also have to keep it together. You see me here, it’s been 5 years now, but you have a partner who is supportive, there’s no reason for you not to pick yourself up.”
	“I want to thank God first and also my son. … I don’t have to ask him for anything. He’s always looking out for me. And he doesn’t care how much money it is, if it’s a lot or not. He’s always willing, and well I thank God for him, because he’s always by my side. Like you see him, he’s here at the meeting and, well, I thank God for my son and for my 3 kids.”
Faith	“And God is already giving [life] to you because look at me: He already has given me 6 years. And I’m going to tell you something that someone told me here. Logically I’ve had these 6 years thanks to my wife. When I first started dialysis, I also got depressed, and you know what he said? ‘From this moment on, you’re on overtime so enjoy it to the max. Live 100%.’”
	“We mustn’t stop because we have this disease. God is allowing us to do now what we didn’t do before. And we must leave behind what we didn’t do right. You must make an effort and push forward. But you must do it with all your might.”
	“I get my strength from God and sometimes I feel sick, but I pray to God. And he gives me strength day by day because otherwise I think I’d be gone now, but God is my strength, yes.”
**Emotional and physical aspects of receiving emergency dialysis**	
Psychosocial and physical distress	“Yes, it’s going to be 5 years for me, but right now in this stage I have in dialysis, I’m going through a stage and I try to be strong and have activities that get me out of that. But I’m having panic and anxiety attacks.”
	“I’ve had several different symptoms. …my jaw would hurt a lot. …when I have to get dialysis and I got a lot of bloody nose spells…and then I would receive dialysis and the symptoms would end. But every week, it’s the same thing all over again.”
	“My potassium was really high yesterday, and the only thing I felt is that I was sitting down like I am right now, and then I couldn’t get up anymore. I got up and I just fell down. …I just seem to lose my strength. I try to get up and just imagine if I was just like a piece of plastic, down I go.”
	“I spent all week lying in bed, and when I got up and I tried to tie my shoes and I couldn’t because I was so dizzy, I started crying. And I have my partner, but depression gets to every one of us. I spent an entire week in bed. And when I got up, I tried to tie my shoes and I couldn’t, and I just started crying, and my wife is here and she saw everything. I was so desperate and angry because I couldn’t find my shoes, and I’m not a person who is used to not doing my own things.”
Mixed experiences with language-concordant care	“Like [I] fear what they might tell me. And also because what I don’t know is how can I also talk if no one speaks Spanish and that’s… how will I tell them?”
Emotional exhaustion from end-of-life conversations	“Is [palliative care] meant to [provide] support or to scare? Because it has already happened to me, I think it must be, like, the doctor who came to see me because according to her, I was going to die, and that only served to scare me, because in my mind I was thinking I will die, I will die, and I was just waiting for my time; and I am still alive.”
	“I will die sooner [if I need to fill out an advance directive]…by being nervous thinking about something [like what I want done if near death] when that might not even happen.”
	“We as Hispanic are not used to that, so this paper [medical durable power of attorney document] makes us feel…well, personally, I feel bad, but at the same time it is some support. I feel bad in the sense of, well, let death come and take me, if I can say that.”
	“I agree with getting that kind of explanation when we are sick. But why, when we get out of that trance, don’t they do research on what were the consequences of all that, because it’s traumatizing.”
Gratitude for clinicians	“I know that Dr [deidentified]…and the other doctor are the right people and we can learn from them. What they know, they can give it to us. It’s something nice, they learned it and they’re giving it to us and we must value their time.”
	“No, well, I thank God and I thank life. For the opportunities that you have here, right? This group of doctors and nurses, people who have helped us in every possible way, so she can get this dialysis. So I’m thankful for all that.”
	“We’re very blessed, right. We thank God because we are very blessed here in this country, because if it wasn’t because of the dialysis, he wouldn’t be alive.”
	“I sincerely thank the hospital a lot because I’ve been a step away from death and you rescue me. By God, you rescue me, and that is something I have to be very thankful for. Thank you doctor, for all you do for us. I say this from the bottom of my heart.”

^a^ Verbatim quotes from interviews conducted in Spanish were translated into English by a translation company (Datagain).

### Theme: Camaraderie and Emotional Support From Peers

#### Peer Support Is Vital for People Newly Diagnosed With Kidney Failure

Participants described emotional distress when first learning of their diagnosis because they did not understand their diagnosis, prognosis, or what it would mean to receive only emergency dialysis. One participant said, “I was devastated when they told me about my kidneys. I wanted to die. …I think someone newly diagnosed with kidney failure must be encouraged.” Participants also described the importance of sharing about symptom accumulation leading up to emergency dialysis with those who were newly diagnosed so they could “learn the ropes.”

#### Safe Space to Build Relationships and Share Hardship With Peers

Participants described that it was difficult to get to know each other in the inpatient dialysis center setting, while the peer support group setting allowed for more personalized connections and relationship building. One participant said, “When you are sitting down at a dialysis chair, you really don’t get to interact much with others compared to getting to know each other at these meetings.” Participants also felt camaraderie and the importance of a safe space for sharing personal hardship and learning from each other about their illness. They described challenges in sharing their physical symptoms and anxiety about death with family. One participant said, “it can be difficult…to talk to your kids because you don’t want your kids to become very depressed. They might try to commit suicide or do poorly in school.” Another participant said, “it’s important to ask questions in a way that you don’t feel judged. …here, no one will scold you.”

#### Hospital Setting for Peer Support Is Ideal

Participants described feeling physical distress after having “so much fluid removed” after 7 days without dialysis. Meeting in the hospital setting was ideal for participants who were physically stressed and needed to cope with emergency dialysis. One participant said, “It was a good way to interact with people because it helped me cope with the stress from dialysis. …after getting done with dialysis, you don’t always feel your best. …I really liked getting together with other people who are going through this.”

#### Solidarity to Survive and Change Policy

Group members supported each other in advocating for receipt of outpatient dialysis and reducing financial burdens by obtaining private health insurance. Participants decided they would fundraise and use the money to support those who needed health insurance. One participant said, “we don’t have money to pay for medication…but we all have to work. …we can donate and receive according to our finances. This is what this group is about—if we unite, we can get through this.” The group also developed a messaging application (WhatsApp) and social media (Facebook) group for members and their families to communicate about fundraising, social resources, and advocacy.

#### Sustainability of Peer Support Group

Participants expressed enjoying group sessions and made plans to spend time together outside the hospital setting. When the study was coming to an end, participants formalized the peer group by developing a nonprofit organization called Club Riñones Latinos (Latino Kidney Club). One participant said, “we want to make a group because we all have kidney issues and not a lot of information. …motivating ourselves is the best strategy.” Another participant said, “We have in mind to create a group, but we don’t know how to build relationships with funders. …we wouldn’t be talking about creating this group if you hadn’t brought us together.”

### Theme: Solutions to Improve Care and Resilience

#### Self-advocacy

Self-advocacy was discussed during every meeting as a means for participants to improve their care. For example, participants discussed downloading a phone translation application so that they could ask clinical care–related questions. They also shared thoughts about the “chain of command” and understanding which individuals they could access if they needed to address a hospital issue. They spoke about avoiding physicians who treated them badly. They noted the importance of knowing one’s body and being adamant when dialysis needed to be stopped to avoid excess fluid removal.

#### Self-motivation and Optimism

Participants described that staying healthy was a personal decision because individuals can choose to believe that they are going to die or live a long life. One participant said, “I haven’t been doing this for long, but…when they started me on dialysis…a lot of people said I was going to die. But kidney failure will not kill you if you don’t want it to. You can be okay if you want. You can live a lot of years. …it’s all up to you.”

#### Improve Kidney Disease Education

Participants described learning about kidney disease on their own. Some participants formed close relationships with their clinicians and asked questions about their kidney disease. One participant said, “There’s a point that I think is very important: to have a very open communication with your doctor. I have done this, and thank God, because she’s helped me a lot.” They also talked about the importance of being honest with their dietitians about culturally traditional foods so that they could receive clarity on dietary restrictions.

#### Emotional Support From Peers and Caregivers

Participants described that their family, caregivers, and peers provided support and motivation to stay healthy. With respect to peer support, one participant said, “I was feeling very depressed at the beginning, and then once I was going through the program and attending the meetings, it was giving me more hope.” With respect to family, 1 participant said, “I think it’s also having a partner that says, ‘Hey, how are you? Snap out of it!’ Or having someone that asks you, ‘How do you feel?’”

#### Faith

Faith played a major role among participants in coping with emergency dialysis. Participants described praying often and using their faith to foster resilience and gratitude for life. One participant said, “I have a lot of faith in God, and I pray a lot. …I understand that everything happens for a reason and that everything happens on God’s time. …God is going to give me time to meet my grandkids and spend time with them.”

### Theme: Emotional and Physical Aspects of Receiving Emergency Dialysis

#### Psychosocial and Physical Distress

Participants described feelings of depression and weekly anxiety from symptoms. One participant said, “I get a panic attack because I don’t want to die. …I think that if I go to sleep that I won’t wake up again.”

#### Mixed Experiences With Language-Concordant Care

Participants had varied experiences receiving language-concordant care, including having to self-advocate for an interpreter, working with interpreters who did not interpret well, and asking for Spanish educational materials after receiving them in English. One participant said, “there are good and not-so-good interpreters. …There are interpreters who say whatever they want and I’ve had to say, ‘Excuse me, I didn’t say that.’”

#### Emotional Exhaustion From End-of-Life Conversations

Participants reported feeling emotionally weighed down by weekly conversations about end-of-life care and advance directives. These conversations took place weekly because they were part of the hospital admission history and physical. Participants reported that these conversations exacerbated their sense that death was imminent. They also described that talking about or making plans for death were taboo in their culture, and they wondered if their clinicians were discussing end-of-life care because they wanted the participant to die. One participant said, “when clinicians talk about advance directives and end-of-life [care], I’m not sure if they want to inform me or scare me. ...is this the doctor’s way out of responsibility because they want us to die?” Others said they felt it was a good idea to be prepared and make plans early.

#### Gratitude for Clinicians

Participants expressed gratitude toward clinicians with whom they had built close relationships during weekly hospital admissions. Participants said they were aware that clinicians and the hospital were required to provide emergency dialysis rather than standard outpatient dialysis because of state policies. Participants also expressed gratitude for emergency dialysis, stating that in their home countries, they would have died because emergency dialysis was not offered.

## Discussion

In this single-group, prospective qualitative study, we found that a peer support group support intervention for undocumented immigrants with kidney failure receiving emergency dialysis was feasible and acceptable. To our knowledge, this is the first prospective peer support group intervention for this population and so may represent a novel strategy to provide emotional support to a uniquely marginalized community whose members face mistrust and discrimination and lack health care coverage.

Peer support may represent a powerful tool associated with an improved illness experience among undocumented immigrants receiving emergency dialysis. In previous studies of patients experiencing chronic kidney disease, peer support was associated with improved self-efficacy and emotional well-being, increased aid in treatment decisions, and increased help for patients developing coping strategies to manage their conditions.^23,24^ For people with kidney disease who are from minoritized racial and ethnic backgrounds, peers with similar cultural backgrounds and lived experiences have been found to be an important source of support.^7,19,25^ In 1 study^26^ using culture-concordant peer support for Latinx individuals with breast cancer, participants reported that peer support helped them express their feelings, feel less anxious and depressed, learn to ask questions, and express their needs to health care professionals and made them feel that someone understood their experience. A peer-led and culturally tailored support group intervention for Mexican-American individuals with type 2 diabetes was associated with increased empowerment and self-efficacy among participants.^27^ Our data suggest the potential value of peer support for improved depression and anxiety, as well as the provision of emotional support through sharing of lived experiences, self-advocacy, and coping mechanisms.

Participants in our study shared struggles with emergency dialysis, such as anxiety and depression, and encouraged each other to discuss these issues with their families and clinicians. Individuals encouraged each other to advocate for themselves while hospitalized by asking for interpreters or knowing their dry weight to avoid sequelae of hypotension in dialysis. Moreover, participants discussed their awareness of the injustice of not being able to receive optimal treatment owing to their immigration status; however, they also said they felt gratitude for the care received because dialysis was not available in their home countries.

An important finding from our study was the use of peer support to promote self-advocacy with policymakers. After this study, participants organized themselves and engaged in research and advocacy with the study team (L.C. and C.C.). Participants formed the Latino Kidney Club, and they developed their own social media and messaging application groups to communicate about advocacy, fundraising, and social resources. Additionally, many participants partnered with L.C. and C.C. to engage in community-based research and to publish studies finding worse outcomes with emergency dialysis.^14,15^ In 2019, a coalition of patients and partners (eg, clinicians, community organizations, and policy leaders) successfully expanded access to standard outpatient hemodialysis for undocumented immigrants with kidney failure through a billing change in Emergency Medicaid.^9,28^ Colorado became the twelfth state to explicitly expand health care coverage of standard dialysis through Emergency Medicaid; 4 years later, 20 states provided statewide health care coverage of standard outpatient hemodialysis.^29^

This study may have key implications for patients with chronic disease who are members of marginalized populations. Other studies examining the role of peer-to-peer mentoring for patients receiving dialysis found that patients receiving dialysis who were mentored had fewer missed dialysis treatments, improved end-of-life discussions, and increased self-efficacy and social support.^30,31,32^ Implementing a peer support program requires identifying, educating, and supervising peer mentors; a 2023 study^33^ found that this was feasible, and multiple organizations, including the National Kidney Foundation and Centers for Medicare & Medicaid Services, offer peer-mentoring services for patients receiving dialysis.^34,35,36^ However, challenges remain, including engagement of patients receiving mentoring and consistent training for mentors. Peer mentorship may also be held outside the dialysis center, allowing for greater privacy, but would require modifying the workflow of the dialysis unit. For undocumented immigrants receiving emergency dialysis, peer support offered a source of companionship in our study. A future randomized clinical trial of peer support may investigate improved depression, anxiety, and emotional resilience through sharing coping mechanisms. Lastly, our findings suggest that peer support may be a powerful tool to promote self-advocacy and patient engagement in treatment choices.

### Limitations

This study has several limitations. Our work included a small sample size and recruited from a single hospital system; therefore, our findings may not be generalizable. Policies for emergency hemodialysis vary by institution, and there may be differences in patient lived experience in access to dialysis care. Furthermore, all study participants were Latinx; therefore, their illness experience cannot be generalized to other racial and ethnic groups. An additional limitation of this study is that race was not collected. Latinx individuals self-report being of any race. Although race is a social construct, it is important to collect race data because systemic and structural racism are pervasive and fundamental causes of health disparities through discrimination and unequal distribution of resources, wealth, and power. According to the US Census, Colorado Latinos as a group self-report as 1.7% American Indian, 3.6% Asian, 4.7% Black, and 86.5% White.^37^

## Conclusions

In this qualitative study, undocumented immigrants with kidney failure who relied on emergency dialysis described wanting to formalize a peer support group because they reported camaraderie and learned strategies to improve their resilience, including self-advocacy and optimism. Our results suggest that group peer support may be feasible and acceptable; it may also provide a patient-centered strategy to address the need for depression, anxiety, and social support services among patients with kidney failure, especially for socially marginalized, uninsured populations whose members report limited English proficiency.

## Notes

Supplement 1. **eTable.** Patient-Selected Meeting Topics

Supplement 2.
Data Sharing Statement

## References

[1] US Renal Data System. 2020 Annual data report. National Institute of Diabetes and Digestive and Kidney Diseases. Accessed May 16, 2023. https://adr.usrds.org/2020

[2] Purnell TS, Bae S, Luo X, . National trends in the association of race and ethnicity with predialysis nephrology care in the United States from 2005 to 2015. JAMA Netw Open. 2020;3(8):e2015003. doi:10.1001/jamanetworkopen.2020.15003 32852554 PMC7453308

[3] Purnell TS, Luo X, Cooper LA, . Association of race and ethnicity with live donor kidney transplantation in the United States from 1995 to 2014. JAMA. 2018;319(1):49-61. doi:10.1001/jama.2017.19152 29297077 PMC5833543

[4] Shen JI, Chen L, Vangala S, . Socioeconomic factors and racial and ethnic differences in the initiation of home dialysis. Kidney Med. 2020;2(2):105-115. doi:10.1016/j.xkme.2019.11.006 32734231 PMC7380374

[5] Cervantes L, Rizzolo K, Carr AL, . Social and cultural challenges in caring for Latinx individuals with kidney failure in urban settings. JAMA Netw Open. 2021;4(9):e2125838. doi:10.1001/jamanetworkopen.2021.25838 34533567 PMC8449281

[6] Cervantes L, Linas S, Keniston A, Fischer S. Latinos With chronic kidney failure treated by dialysis: understanding their palliative care perspectives. Am J Kidney Dis. 2016;67(2):344-347. doi:10.1053/j.ajkd.2015.09.026 26612276

[7] Cervantes L, Jones J, Linas S, Fischer S. Qualitative interviews exploring palliative care perspectives of Latinos on dialysis. Clin J Am Soc Nephrol. 2017;12(5):788-798. doi:10.2215/CJN.10260916 28404600 PMC5477217

[8] Rizzolo K, Cervantes L. Immigration status and end-stage kidney disease: role of policy and access to care. Semin Dial. 2020;33(6):513-522. doi:10.1111/sdi.12919 33089565

[9] Cervantes L, Mundo W, Powe NR. The status of provision of standard outpatient dialysis for US undocumented immigrants with ESKD. Clin J Am Soc Nephrol. 2019;14(8):1258-1260. doi:10.2215/CJN.03460319 31171588 PMC6682810

[10] Cervantes L, Tuot D, Raghavan R, . Association of emergency-only vs standard hemodialysis with mortality and health care use among undocumented immigrants with end-stage renal disease. JAMA Intern Med. 2018;178(2):188-195. doi:10.1001/jamainternmed.2017.7039 29255898 PMC5838789

[11] Nguyen OKVM, Vazquez MA, Charles L, . Association of scheduled vs emergency-only dialysis with health outcomes and costs in undocumented immigrants with end-stage renal disease. JAMA Intern Med. 2019;179(2):175-183. doi:10.1001/jamainternmed.2018.5866 30575859 PMC6439652

[12] Cervantes L, Rizzolo K, Tummalapalli SL, Powe NR. Economic impact of a change in Medicaid coverage policy for dialysis care of undocumented immigrants. J Am Soc Nephrol. 2023:10.1681/ASN.0000000000000139. doi:10.1681/ASN.0000000000000139 37022116 PMC10356160

[13] Cervantes L, Fischer S, Berlinger N, . The illness experience of undocumented immigrants with end-stage renal disease. JAMA Intern Med. 2017;177(4):529-535. doi:10.1001/jamainternmed.2016.8865 28166331

[14] Cervantes L, Tong A, Camacho C, Collings A, Powe NR. Patient-reported outcomes and experiences in the transition of undocumented patients from emergency to scheduled hemodialysis. Kidney Int. 2021;99(1):198-207. doi:10.1016/j.kint.2020.07.024 33129568

[15] Cervantes L, Carr AL, Welles CC, . The experience of primary caregivers of undocumented immigrants with end-stage kidney disease that rely on emergency-only hemodialysis. J Gen Intern Med. 2020;35(8):2389-2397. doi:10.1007/s11606-020-05696-3 32076974 PMC7403248

[16] Cervantes L, Zoucha J, Jones J, Fischer S. Experiences and values of Latinos with end stage renal disease: a systematic review of qualitative studies. Nephrol Nurs J. 2016;43(6):479-493. 30550077

[17] Cervantes L, Hull M, Keniston A, Chonchol M, Hasnain-Wynia R, Fischer S. Symptom burden among Latino patients with end-stage renal disease and access to standard or emergency-only hemodialysis. J Palliat Med. 2018;21(9):1329-1333. doi:10.1089/jpm.2017.0663 29847204

[18] Fisher EB, Ballesteros J, Bhushan N, . Key features of peer support in chronic disease prevention and management. Health Aff (Millwood). 2015;34(9):1523-1530. doi:10.1377/hlthaff.2015.0365 26355054

[19] Sokol R, Fisher E. Peer support for the hardly reached: a systematic review. Am J Public Health. 2016;106(7):e1-e8. doi:10.2105/AJPH.2016.303180 27196645 PMC4984766

[20] Cervantes L, Hasnain-Wynia R, Steiner JF, Chonchol M, Fischer S. Patient navigation: addressing social challenges in dialysis patients. Am J Kidney Dis. 2020;76(1):121-129. doi:10.1053/j.ajkd.2019.06.007 31515136 PMC8118353

[21] Strauss A, Corbin JM. Basics of Qualitative Research Techniques and Procedures for Developing Grounded Theory. 2nd ed. Sage Publications; 1998.

[22] Corbin J, Strauss A. Basics of Qualitative Research: Technique and Procedures for Developing Grounded Theory. 4th ed. Sage Publications; 2014.

[23] Hughes J, Wood E, Smith G. Exploring kidney patients’ experiences of receiving individual peer support. Health Expect. 2009;12(4):396-406. doi:10.1111/j.1369-7625.2009.00568.x 19691464 PMC5060506

[24] Winterbottom AE, Bekker HL, Conner M, Mooney AF. Patient stories about their dialysis experience biases others’ choices regardless of doctor’s advice: an experimental study. Nephrol Dial Transplant. 2012;27(1):325-331. doi:10.1093/ndt/gfr266 21642512

[25] Walker RC, Walker S, Morton RL, Tong A, Howard K, Palmer SC. Māori patients’ experiences and perspectives of chronic kidney disease: a New Zealand qualitative interview study. BMJ Open. 2017;7(1):e013829. doi:10.1136/bmjopen-2016-013829 28104711 PMC5253593

[26] Nápoles-Springer AM, Ortíz C, O’Brien H, Díaz-Méndez M. Developing a culturally competent peer support intervention for Spanish-speaking Latinas with breast cancer. J Immigr Minor Health. 2009;11(4):268-280. doi:10.1007/s10903-008-9128-4 18340533 PMC3832434

[27] Haltiwanger EP. Effect of a group adherence intervention for Mexican-American older adults with type 2 diabetes. Am J Occup Ther. 2012;66(4):447-454. doi:10.5014/ajot.2012.004457 22742693

[28] Cervantes L, Johnson T, Hill A, Earnest M. Offering better standards of dialysis care for immigrants: the Colorado example. Clin J Am Soc Nephrol. 2020;15(10):1516-1518. doi:10.2215/CJN.01190120 32444395 PMC7536747

[29] Rizzolo K, Dubey M, Feldman KE, Powe NR, Cervantes L. Access to kidney care for undocumented immigrants across the United States. Ann Intern Med. Published online April 25, 2023. doi:10.7326/M23-0202 37094340

[30] St Clair Russell J, Southerland S, Huff ED, Thomson M, Meyer KB, Lynch JR. A peer-to-peer mentoring program for in-center hemodialysis: a patient-centered quality improvement program. Nephrol Nurs J. 2017;44(6):481-496. 29281772

[31] Perry E, Swartz J, Brown S, Smith D, Kelly G, Swartz R. Peer mentoring: a culturally sensitive approach to end-of-life planning for long-term dialysis patients. Am J Kidney Dis. 2005;46(1):111-119. doi:10.1053/j.ajkd.2005.03.018 15983964

[32] “Nephrology News and Issues” highlights peer mentoring study at UVA’s Lynchburg dialysis facility. University of Virginia. Accessed April 17, 2023. https://news.med.virginia.edu/medicinematters/?p=13506

[33] Golestaneh L, Golovey R, Navarro-Torres M, . Feasibility of a peer mentor training program for patients receiving hemodialysis: an educational program evaluation. Kidney Med. 2023;5(5):100630. doi:10.1016/j.xkme.2023.100630 37139080 PMC10149397

[34] National Kidney Foundation of Michigan. Peer mentoring. Accessed April 17, 2023. https://nkfm.org/program-article/peer-mentoring/

[35] End-Stage Renal Disease Network Program. What is the Peer Mentoring Educational Program? Accessed April 17, 2023. https://esrd.ipro.org/patients-family/pfe/peer-mentoring/

[36] Golestaneh L, Melamed M, Kim RS, . Peer mentorship to improve outcomes in patients on hemodialysis (PEER-HD): a randomized controlled trial protocol. BMC Nephrol. 2022;23(1):92. doi:10.1186/s12882-022-02701-1 35247960 PMC8897762

[37] US Census Bureau. QuickFacts Colorado. Accessed May 4, 2023. https://www.census.gov/quickfacts/CO

