# Supplementary material for: Assessment of a Peer Support Group Intervention for Undocumented Latinx Immigrants With Kidney Failure
Source: JAMA Netw Open. 2023 Jun 21;6(6):e2319277. doi: 10.1001/jamanetworkopen.2023.19277 (PMC10285568; doi:10.1001/jamanetworkopen.2023.19277)
Supplement: Supplement 1. — eTable. Patient-Selected Meeting Topics [file jamanetwopen-e2319277-s001.pdf]

Supplemental Online Content

Cervantes L, Rizzolo K, Indovina KA, et al. Assessment of a peer support group intervention for undocumented Latinx immigrants with kidney failure. *JAMA Netw Open*. 2023;6(6):e2319277. doi:10.1001/jamanetworkopen.2023.19277

eTable. Patient-Selected Meeting Topics

This supplemental material has been provided by the authors to give readers additional information about their work.

© 2023 Cervantes L et al. *JAMA Network Open.*

eTable. Patient-Selected Meeting Topics

(1) Mental health<br><br>(2) How to talk to caregivers about kidney disease and emotional support for caregivers<br><br>(3) Permanent vascular access complications<br><br>(4) Kidney diet<br><br>(5) Immigration and legal rights<br><br>(6) Palliative care<br><br>(7) Advance directives<br><br>(8) Symptoms from kidney failure and how to manage them<br><br>(9) How the dialysis machine works<br><br>(10) Sexual health<br><br>(11) How to select and cook healthy foods.

© 2023 Cervantes L et al. *JAMA Network Open.*
