# Supplementary material for: Assessment of a Peer Support Group Intervention for Undocumented Latinx Immigrants With Kidney Failure
Source: JAMA Netw Open. 2023 Jun 21;6(6):e2319277. doi: 10.1001/jamanetworkopen.2023.19277 (PMC10285568; doi:10.1001/jamanetworkopen.2023.19277)
Supplement: Supplement 2. — Data Sharing Statement [file jamanetwopen-e2319277-s002.pdf]

Data Sharing Statement

Cervantes. Assessment of a Peer Support Group Intervention for Undocumented Latinx Immigrants With Kidney Failure. *JAMA Netw Open*. Published June 21, 2023. doi:10.1001/jamanetworkopen.2023.19277

Data
Data available: No

Additional Information
Explanation for why data not available: Small study with 23 people.
